# Unobtrusive Cognitive Assessment in Smart-Homes: Leveraging Visual Encoding and Synthetic Movement Traces Data Mining

**DOI:** 10.3390/s24051381

**Published:** 2024-02-21

**Authors:** Samaneh Zolfaghari, Annica Kristoffersson, Mia Folke, Maria Lindén, Daniele Riboni

**Affiliations:** 1 School of Innovation, Design and Engineering, Division of Intelligent Future Technologies, Mälardalen University, 721 23 Västerås, Sweden; annica.kristoffersson@mdu.se (A.K.); mia.folke@mdu.se (M.F.); maria.linden@mdu.se (M.L.); 2 Department of Mathematics and Computer Science, University of Cagliari, 09124 Cagliari, Italy; riboni@unica.it

**Keywords:** trajectory mining, visual feature extraction, smart environments, machine learning, environmental sensors, ambient sensing, ambient assisted living

## Abstract

The ubiquity of sensors in smart-homes facilitates the support of independent living for older adults and enables cognitive assessment. Notably, there has been a growing interest in utilizing movement traces for identifying signs of cognitive impairment in recent years. In this study, we introduce an innovative approach to identify abnormal indoor movement patterns that may signal cognitive decline. This is achieved through the non-intrusive integration of smart-home sensors, including passive infrared sensors and sensors embedded in everyday objects. The methodology involves visualizing user locomotion traces and discerning interactions with objects on a floor plan representation of the smart-home, and employing different image descriptor features designed for image analysis tasks and synthetic minority oversampling techniques to enhance the methodology. This approach distinguishes itself by its flexibility in effortlessly incorporating additional features through sensor data. A comprehensive analysis, conducted with a substantial dataset obtained from a real smart-home, involving 99 seniors, including those with cognitive diseases, reveals the effectiveness of the proposed functional prototype of the system architecture. The results validate the system’s efficacy in accurately discerning the cognitive status of seniors, achieving a macro-averaged *F*_1_-score of 72.22% for the two targeted categories: cognitively healthy and people with dementia. Furthermore, through experimental comparison, our system demonstrates superior performance compared with state-of-the-art methods.

## 1. Introduction

As we witness, the number of older adults in relation to people of working age is steadily increasing. According to [1], there were an estimated 258 million people aged over 65 globally in 1980, a number that surged to 771 million in 2022. Projections indicate that the older adult population will reach 994 million in 2030 and 1.6 billion in 2050. This demographic transition is expected to lead to a rise in the old-age dependency ratio within the European Union, from 27.5% in 2013 to a projected 49.4% in 2050. Associated with this societal shift is an increased number of people with declining cognitive function and mobility, which has significant societal consequences [2]. Notably, the global number of people with dementia (PwD) exceeded 35 million in 2013 and is anticipated to double in 2030, reaching a staggering 115 million in 2050 [3]. Therefore, there will be an amplified demand for professional caregivers, particularly for individuals with chronic conditions like dementia and cognitive impairment. These individuals face increased risks of diminished independence and safety concerns because a significant portion of those in need of care prefer to remain in their own residences. Consequently, early detection of cognitive impairments could be pivotal in facilitating timely therapies and enabling extended periods of independence and social engagement [4,5]. Hence, a promising strategy involves focusing on the demographic transition and addressing the aging trend by shifting from formal care settings to in-home care.

With the advancement of sensor-based technologies and AI algorithms, cutting-edge e-health solutions are emerging for in-home care. These solutions target the prevention of chronic diseases and facilitate time-dependent and location-based monitoring, enable the extraction of hidden information, and allow the management of older adults’ behaviors through a variety of assistive technologies [5,6,7]. Various sensor-based systems, including wearable, vision-based, and environmental sensors, aim to detect early signs of cognitive impairment by monitoring behavioral patterns. Some rely on clinical models [8,9], while others use pattern mining methods [10]. Nevertheless, methods able to detect performed activities raise privacy concerns and may exhibit reduced accuracy, attributed to potential errors in activity recognition modules [11]. To overcome these challenges, a promising solution involves leveraging positioning technologies and trajectories for monitoring the movement patterns of older adults instead of personal activity [6,12,13,14,15].

**Figure 1:**
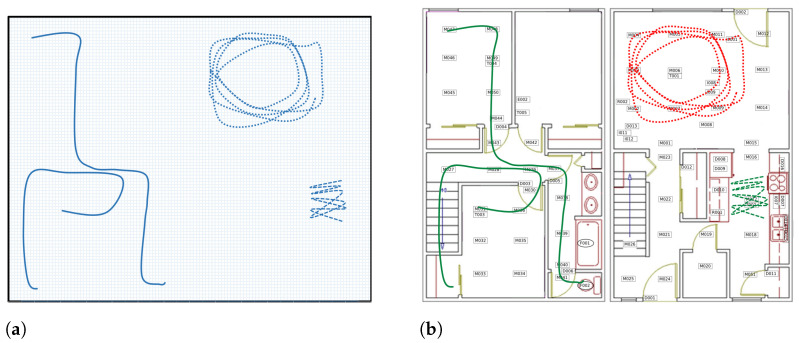
Example of movement patterns based on the Martino-Saltzman model, adopted from [18]. In (**a**), all four trajectories seem abnormal without context. Solid lines resemble direct and random walks, the dashed line indicates pacing, and the dotted line suggests a lapping movement. Within the home floor plan context (**b**), only one trajectory is likely abnormal. Green solid lines represent the shortest paths, the back-and-forth movement in the kitchen (green dashed line) might be a normal table-setting activity, while the repeated looping movement in the living room (red dotted line) could indicate an abnormal lapping pattern, possibly linked to cognitive issues.

Movement-based indicators serve as established metrics for classifying abnormal movement patterns exhibited by PwDs [5,12]. Initially, Algase et al. [16] introduced the concept of locomotion to describe the temporal phases of movement. In this context, a locomotion episode is defined as a rhythmic sequence comprising walking and non-walking phases. A widely recognized model in this framework was introduced by Martino-Saltzman [17]. This model classifies trajectories into four distinct patterns of movement: direct, random, lapping, or pacing. Cognitively healthy (CH) individuals typically follow a direct path, whereas random, pacing, and lapping patterns are characteristic indicators of dementia. An example of these locomotion patterns in a smart-home context is represented in Figure 1.

Notably, research findings indicate that individuals with severe dementia display movement-based anomalies throughout the day, whereas those with moderate dementia exhibit an increased percentage of such anomalies, particularly in the evening and predominantly at night [5,17].

Existing efforts to identify cognitive impairment through movement patterns are predominantly centered on outdoor environments. Lin et al. [19] analyzed GPS trajectories in outdoor settings to detect wandering using the Martino-Saltzman model [17]. Ng and Kong [20] devised a smart GPS tracker for the secure outdoor mobility of the elderly, integrating wandering detection and activity recognition. However, since many elderly individuals, particularly those experiencing cognitive decline, spend a significant portion of their time indoors, assessing cognitive status using indoor movement traces becomes crucial. Identifying indoor wandering poses significant challenges due to the intricacies of navigating within smart-home layouts. The presence of obstacles and the need to navigate around furniture, doorways, and other objects can impede movement patterns. Additionally, the execution of daily activities within the home can further complicate the analysis, as activities such as cooking, cleaning, and interacting with household items may influence the trajectory of movement. These factors contribute to the complexity of accurately identifying and interpreting indoor wandering behaviors.

Previous works have addressed this challenge. Vuong et al. [21] applied supervised machine learning (ML) to categorize indoor trajectories according to the Martino-Saltzman model [17]. Lin et al. [22] introduced a method for identifying repetitive indoor movement traces episodes based on established wandering models. Khodabandehloo and Riboni [23] proposed a collaborative mining approach using statistical features from indoor trajectories to assess cognitive status. Kearns et al. [15] utilized precise localization technologies within a retirement home. They gauged movement tortuosity as a means to identify wandering episodes, and discovered a correlation between increased path tortuosity and lower Mini-Mental State Examination (MMSE) scores, as evaluated by clinicians. Subsequent studies revealed additional predictive features such as speed, path-efficiency, and turn-angle for dementia [13]. Faruk et al. [24] explored the same approach proposed in the previous work of Zolfaghari et al. in [12]. They employed movement traces encoded into images, collected within a smart indoor environment, and utilized a convolutional neural network (CNN). While deep learning methods have demonstrated high recognition rates, their effectiveness is contingent upon the availability of substantial labeled training data. This poses challenges in real-world settings, particularly within sensitive domains such as the one addressed in [24]. Other studies demonstrated a significant correlation between in-home walking velocity, activity patterns, and the inhabitant’s cognitive status [14].

Furthermore, Zolfaghari et al.’s earlier studies in [12,25] critically assessed their limitations and identified crucial areas for improvement. This includes the imperative to enhance the research by incorporating additional features, suggesting a more comprehensive approach to feature enrichment. In the domain of spatial tracking, the recommendation is to move beyond reliance on passive infrared (PIR) and door sensors, advocating for the adoption of more precise radio-frequency identification (RFID)-based localization technologies to refine spatial data accuracy. Additionally, recognizing an imbalance in class distribution within the general population, the exploration of imbalanced classification techniques was initiated and discussed in [26]. These insightful limitations not only shaped the trajectory of the current research but also laid the foundation for the advancements presented in this article.

Our current study tackles the aforementioned challenges while also addressing a significant research gap in the field. While existing methodologies mainly rely on numerical feature extraction from locomotion data [13,15,23], we introduce a novel approach by utilizing image-based techniques. This innovative method allows us to identify abnormal indoor movement patterns, which potentially serve as indicators of cognitive decline.

We employed publicly available data obtained from a real smart-home and developed an unobtrusive sensor-based trajectory mining system. This system transforms movement traces within an indoor environment into interpretable images, incorporating visual cues associated with speed of movement, sensor activations, and interactions with objects into the image-encoding process. This innovative approach was inspired by the earlier works of Zolfaghari et al. in cognitive assessment, as presented in [12,26], as well as their contributions to human activity recognition outlined in [27].

This article distinguishes itself from prior studies [6,12,26,28] by visualizing movement pattern through images, discerning interactions with objects on a floor plan representation of the smart-home by additional visual cues and employing different image description features specifically designed for image analysis tasks, namely Connected Region Features (CRF) [29] and Speeded-Up Robust Features (SURF) [30]. We employ a singular trajectory image, as opposed to two. Existing studies have laid the foundation, but our approach surpasses them by offering flexibility through the effortless incorporation of additional features derived from sensor data. This adaptability enhances the system’s robustness and makes it well-suited for real-world applications.

A notable aspect of this approach is the dependence not only on PIR sensors and door sensors but also on RFID sensors integrated into everyday objects, ensuring an unobtrusive and privacy-preserving system, while incorporating more accurate localization technology. The study addresses the scarcity of large-scale sensor-based datasets for cognitive impairment evaluation, highlighting the challenge of collecting data from PwDs, leading to an imbalanced scenario. In contrast, acquiring data from CH individuals is comparatively straightforward [5]. Consequently, the classification process is enhanced using the synthetic minority oversampling technique (SMOTE) [31] to address imbalanced conditions.

We developed a functional prototype of the system architecture and conducted extensive analysis with real-world data acquired from a real smart-home dataset involving 99 older adults. In summary, the contributions of this article include:

- A novel visual encoding method for cognitive assessment;
- Employing different image features designed for image analysis tasks;
- Utilizing synthetic data generation to enhance the model performance;
- A functional prototype of an unobtrusive system architecture;
- A comprehensive experimental evaluation.

Our approach tackles crucial challenges in adaptability and model generalization by employing data augmentation and enhancing the representation of trajectories from the minority class. This contributes to a more balanced and robust dataset for our cognitive assessment framework. Furthermore, the incorporation of additional features bolsters and enriches the comprehensiveness of our approach. These supplementary features not only encode rich information from sensor events but also enhance model generalization, leading to improved performance.

The remainder of this article is structured as follows. Section 2 illustrates our overall system architecture. Section 3 reports on the system setup and experimental evaluation. Finally, Section 4 discusses the results and the limitations of this work, concludes the article, and outlines future directions.

## 2. Methodology

**Figure 2:**
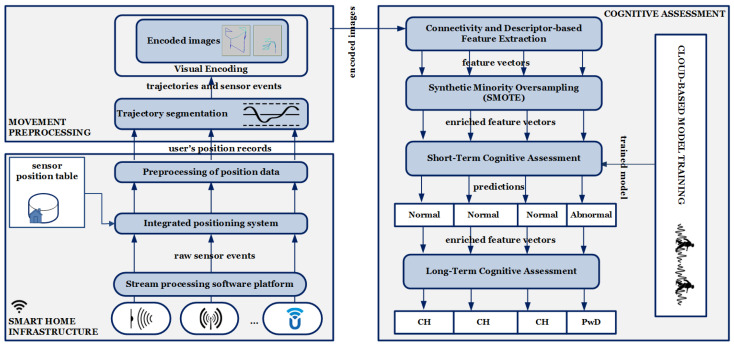
Overview of the functional prototype of the system architecture.

This section provides an overview of the functional prototype of the system architecture, which is depicted in Figure 2. We will examine each module and its sub-modules in more detail.

### 2.1. Overview of the Functional Prototype of the System Architecture

The architecture is built upon a smart-home infrastructure that leverages a stream processing software platform, an integrated positioning system, and pre-processing of position data. This integration enables the synthesis of sensor data and extraction of spatio-temporal information from events based on the relative position of each sensor in the home, as well as the implementation of noise reduction, data cleaning, and partitioning the temporal stream of position records into trajectories. Within the movement preprocessing module, the trajectories are utilized to encode movement patterns and object interactions. This is achieved through trajectory segmentation and visual encoding, resulting in trajectory images.

These encoded images undergo feature extraction using connectivity and descriptor-based feature extraction, and the output is feature vectors including distinctive information from trajectory images, followed by SMOTE to enhance the minority class, i.e., PwDs. These feature vectors are processed through traditional ML algorithms in the short-term cognitive assessment. This process categorizes each trajectory, distinguishing whether it corresponds to a CH individual with normal movement patterns or a PwD with abnormal movement patterns. The concluding sub-module within the cognitive assessment module is the long-term cognitive assessment, tasked with formulating a diagnostic hypothesis for the individual. This hypothesis may indicate either CH or PwD.

In the study presented in this article, we focus on the scenario where an individual resides alone at home, a prevalent situation among elderly people. To accommodate seniors living with others or pets, our system can be readily expanded by integrating an identity-aware positioning system or incorporating a data association algorithm. This algorithm is responsible for linking each location reading to the specific individual who triggered the corresponding sensor, as discussed by Riboni et al. in their work [32]. In the rest of this section, we explain the mentioned modules and sub-modules in detail.

### 2.2. Smart-Home Infrastructure

In this study, we employed publicly available data obtained from a real smart-home, as collected by the Center of Advanced Studies in Adaptive Systems (CASAS) at Washington State University (WSU), Pullman, WA, USA (note: http://casas.wsu.edu/datasets/assessmentdata.zip (accessed on 25 September 2023)) [33].

The CASAS smart-home is a two-story apartment equipped with a range of sensor types, incorporating both ambient and wearable variants. Among these sensors are PIR motion detectors, used to pinpoint an individual’s location within the house, door sensors (open/closed), RFID-embedded sensors in kitchen items for tracking interactions with objects and their usage, power sensors for detecting the operation of specific electrical appliances, and temperature sensors, among other functionalities. These sensors are responsible for the continuous data collection. The smart-home layout encompasses a living/dining room, three bedrooms, a kitchen, and a bathroom. Further details about this dataset can be found in [33].

Since the central focus of this study is on processing movement data, the foundational methods of our contribution remain largely independent of the specific sensor infrastructure in place. Consequently, in this study, we employed a publicly available sensor-equipped smart-home infrastructure capable of real-time monitoring of the smart-home resident’s position by an array of ambient sensors, such as PIR motion sensors and door sensors, and interactions with everyday objects and appliances with RFID-embedded sensors. Indeed, the positioning infrastructure comprised 52 PIR motion sensors attached to the ceiling, providing an approximate spatial resolution of one meter. It is worth noting, however, that there is no available information regarding the sampling rate.

Given our assumption that the residence was occupied by a unique individual, our focus was not directed towards linking the sensor record to the specific person who activated it. The researchers of CASAS acquired data from 400 adult subjects aged over 18 who underwent comprehensive clinical assessments for cognitive health. Subjects were categorized into 10 diagnosis groups. Since our focus was on assessing cognitive issues, we considered data from 99 subjects, including 80 CH older adults aged 60 to 74 and 19 PwDs capable of performing home activities. Each person was monitored for only a few hours on a single day. It should be noted that the age group of the PwDs is not provided in the CASAS project [33].

#### 2.2.1. Stream Processing Software Platform

The Stream Processing Software Platform in our proposed functional prototype of the system architecture was developed to collect raw sensor events. Each time a sensor is triggered, the platform transmits a **raw sensor event**, e=〈t,s_id,v〉, to a stream processing software platform (e.g., Apache Kafka) for integration and temporal synchronization, where, *t* represents the timestamp of the event, s_id denotes the unique identifier of the sensor, and *v* signifies the generated value. For reference, an example of raw sensor events is illustrated in Table 1.

#### 2.2.2. Integrated Positioning System

The integrated positioning system was developed to gain spatio-temporal insights effectively from sensor events by leveraging the relative positioning of each sensor within the smart-home. The sensor position table serves as a repository for the spatial coordinates of each sensor within the smart-home. Each entry in this table takes the form of a triple:

〈s_id,(x,y)〉,

where s_id is the unique identifier of the sensor, and (x,y) denote the respective relative coordinates of that sensor in the smart-home layout. For reference, an example of a sensor position table is illustrated in Table 2.

Upon receiving a sensor record, the positioning system combines this record with the data from the position table, subsequently extracting and recording the coordinates alongside the timestamp value, resulting in a **position record**, *p*:

p=〈x,y,t〉,

where *x* and *y* represent the relative spatial coordinates of the smart-home resident at timestamp *t*.

#### 2.2.3. Pre-Processing of Position Data

The pre-processing of position data is pivotal for noise reduction and refining data, especially in real-world scenarios. To achieve this, we developed two noise-reduction techniques, as detailed by Zolfaghari et al. in [12]. On the position history, *P*, two key steps were taken: firstly, excluding $p_{i+1}$ from *P* if the speed between consecutive position records $\langle p_{i},p_{i+1}\rangle$ exceeded a defined threshold *v* (set to 15 m/s in our experiments based on typical speed ranges); secondly, removing $p_{i+1}$ from *P* if the distance between consecutive position records exceeded a threshold *d* (set to 5 m in our experiments, considering sensor layout). These measures collectively enhanced the quality of position data for subsequent analysis. The speed range might seem too high when talking about people moving in an apartment. This is because the CASAS smart-home uses a positioning system with around a 1-meter accuracy, and the sensors’ detection ranges overlap. So, the calculated speed is an estimate and might have errors. Despite this, our experiments show that this estimated speed value is still helpful in making our functional prototype of the system architecture work better.

### 2.3. Movement Preprocessing

This module is in charge of trajectory segmentation and visually representing the salient features of each trajectory through trajectory images. Following this, we will provide a detailed explanation of each respective sub-module.

#### 2.3.1. Trajectory Segmentation

A trajectory, representing a single episode of movement, is a connected sequence of temporally linked positions. We utilized non-overlapping segmentation methods to represent movement patterns precisely. In essence, a trajectory comprises movement and non-movement phases. Non-movement phases occur between consecutive sensor activations, not exceeding a given threshold, $T_{s}$. We varied $T_{s}$ from 60 s to 480 s in the experiments.

The segmentation algorithm discerns non-movement phases by detecting intervals where no sensor is triggered for more than $T_{s}$. If there is no user movement detected for more than $T_{s}$, it signals the conclusion of the previous trajectory and initiates a new one. This method supports the identification of essential locations and events, contributing to thorough behavioral analysis, enhancing data interpretation, and improving abnormal movement detection. The thorough details regarding the trajectory segmentation strategy can be found in [12].

#### 2.3.2. Visual Encoding

To enhance trajectory classification and cognitive assessment, this sub-module is in charge of visually encoding segmented trajectories and events of interest (EOI) in images, inspired by the method presented in [27]. In this context, EOIs refer to object interactions, the resident’s position, and movement indicators within the smart-home, which can be gathered through the sensor events. These aspects contribute to the interpretability of the generated images, enabling a clearer understanding of movement patterns, interactions, and activities within the smart-home environment. Our encoded images offer enhanced interpretability compared with other visual encoding methods, such as the one proposed by Gochoo et al. [6]. In their approach, trajectories are represented as binary images, with the *x*-axis denoting the temporal order of sensor activation and the *y*-axis representing sensor identifiers. While their method employs deep convolutional neural networks for classification, the transformation from two-dimensional to one-dimensional space in their approach results in partial disruption of spatial information. This discrepancy arises from the mapping of three-dimensional spatio-temporal trajectory points onto a two-dimensional grid, where metric operations and topological relationships are not preserved. Consequently, proximity in geographic space may not translate to proximity in the grid-based representation, affecting the accuracy of pattern recognition.

Table 3 reports EOI features considered in our experimental setup (Section 3) with their respective colors. It should be noted that, in the CASAS dataset [33], motion sensors are denoted by ‘M’, door sensors by ‘D’, and RFID-embedded sensors by ‘I’.

**Figure 3:**
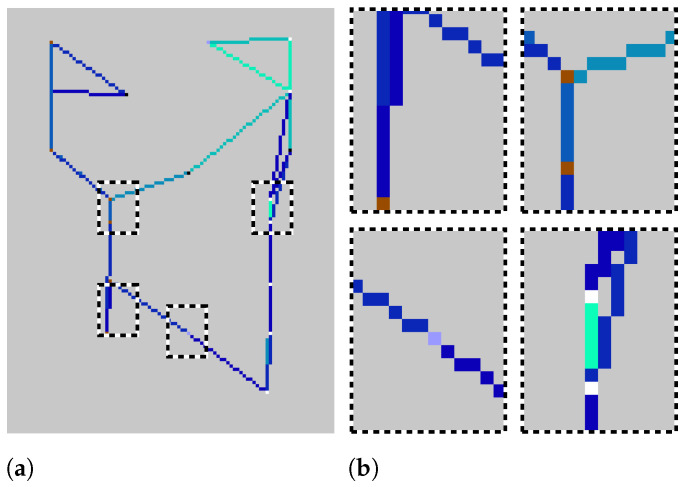
(**a**) Illustration of an encoded image showcasing directional changes. Dashed boxes in black and white highlight regions of interest, each zoomed in on the right. (**b**) Zoomed views reveal details within the boxed areas. *White points* indicate right-side directional changes, *Brown points* indicate left-side directional changes, and *Vivid violet points* indicate movement towards the central position. The shade level of the *Blue line* depends on the speed of the movement.

The Sensor Position category in Table 3 is employed to signify directional changes and is distributed across the smart-home in three directions: left-side (brown points), center-ward (vivid violet points), and right-side (white points). These marked positions in the trajectory image offer insights into the spatial distribution of sensors within the smart-home. The Sensor Interaction category indicates interactions with doors and used objects, proving highly beneficial for identifying specific activities. In the Movement Indicators category, a red point along the trajectory indicates a moment when the resident remains stationary for over 2 seconds. Black points represent sharp angles, equal to or exceeding 90 degrees, signifying abrupt changes in the trajectory’s direction. Furthermore, as depicted in Figure 3, the resident’s trajectory in the smart-home is illustrated by a blue line, with varying shades denoting different movement speeds. Lighter shades indicate an increase in speed, while darker shades signify a decrease in the speed of movement.

It is important to note that, in our smart-home environment, each pixel corresponds to an area of approximately 0.1 m^2^. This spatial resolution results in image dimensions of 100 by 130 pixels in the RGB color model. The line thickness in the visual representation signifies the frequency of traversal for each path, with a weight of 1 assigned for a single traversal, and this weight increasing with subsequent traversals. Consequently, paths that are frequently traveled appear more pronounced in the visualization. In instances where there are multiple traversals within a single trajectory, the image reflects the most recent speed data for that specific path, ensuring that the representation accurately captures the latest speed information.

### 2.4. Cognitive Assessment

The Cognitive Assessment module is responsible for extracting vision-based features, enhancing the representation of trajectories by SMOTE, and conducting both short-term and long-term cognitive assessments to determine if the walked path is made by a CH individual or someone with dementia. Following, we will provide a detailed explanation of each respective sub-module.

#### 2.4.1. Connectivity and Descriptor-Based Feature Extraction

Image descriptors are methods used to capture distinctive information from images, and in this specific work they refer to evaluating CRF [29] and SURF [30]. Indeed, we chose CRF and SURF for our image-encoding method due to their effectiveness in image analysis tasks, complementarity in capturing different aspects of the image, and compatibility with existing systems. CRF provides valuable spatial information by capturing connected regions, while SURF is robust to changes in scale, rotation, and lighting conditions, making it suitable for detecting local image features. Leveraging these features, we aimed to create a comprehensive representation of trajectory images to identify abnormal movement patterns in smart-home environments.

To extract these features, in addition to having RGB trajectory images, we initially converted our trajectory images into both binary and grayscale formats. In binary images, pixels with luminance greater than the specified threshold (thr=0.5) are replaced with the value 1 (white), and all other pixels are set to the value 0 (black). Meanwhile, the process of converting RGB images to grayscale involves eliminating hue and saturation information while preserving luminance.

The CRF features analyze properties of regions of pixels in an image, where a region is defined as a group of connected pixels. These features are employed to analyze regions of interest in images [29]. In this case, they are encoded movements. In order to extract these features, we used the ‘regionprops’ function in MATLAB R2023a (note: https://it.mathworks.com/help/images/ref/regionprops.html (accessed on 10 October 2023)), which measures properties of image regions in a binary image and the corresponding grayscale image. Notable features in this category include:

- *Area*: this represents the total number of pixels that compose the trajectory path or shape. Each pixel within the region contributes to the overall area. It includes all pixels within the defined region, regardless of their intensity or value.
- *MajorAxisLength*: this represents the length of the longest diameter of the ellipse that best fits the trajectory region. It measures the elongation of the trajectory: a longer MajorAxisLength indicates a more stretched shape, while a shorter one suggests a more compact or circular trajectory.
- *MinorAxisLength*: in a trajectory image, this is the length of the shorter diameter of the ellipse that best fits the trajectory region. It measures the width or thickness of the trajectory, with a longer MinorAxisLength suggesting a wider trajectory and a shorter one indicating a narrower shape.
- *Eccentricity*: this measures how stretched or elongated the trajectory is when represented by an equivalent ellipse. It is a dimensionless value ranging between 0 (perfect circle) and 1 (infinitely elongated). Higher eccentricity suggests a more elongated trajectory, while lower values indicate a more circular shape. This metric provides insights into the overall shape of the trajectory.
- *Orientation*: in a trajectory image, this indicates the angle between the x-axis and the major axis of the ellipse that best fits the trajectory region, providing insights into the directional alignment of the trajectory.
- *Perimeter*: this measures the total length of the boundary of the trajectory region, offering information about the complexity or irregularity of the trajectory path.
- *Circularity*: this is a metric that quantifies the roundness of the trajectory region by considering the relationship between its area and perimeter, and is computed as $\left(4*\pi *Area/Perimeter^{2}\right)*{\left(1-0.5/r\right)}^{2}$, where r=Perimeter/(2∗π)+0.5.
- *FilledArea*: the number of ‘on’ pixels (i.e., pixels with a value of 1) within the image. It represents the total number of pixels that forms the trajectory path or shape. This metric provides information about the spatial coverage or size of the trajectory within the image.
- *EulerNumber*: this offers valuable insights into the topological features of the trajectory region, facilitating a deeper understanding of the trajectory’s structural complexity. The calculation involves discerning the disparity between the number of objects and the presence of “holes” within the region. In this context, “holes” refer to areas within a binary image enclosed by connected boundaries but distinct from the main object. These regions represent empty spaces or voids within the primary object or shape. When computing the EulerNumber, the process entails subtracting the count of holes from the number of connected components or objects. Positive Euler values indicate the presence of voids, implying a more intricate and complex structure, whereas negative values suggest a more cohesive or compact arrangement without such openings.
- *EquivDiameter*: the diameter of a circle with the same area as the trajectory region. It offers a representative size measure for the trajectory, providing a single-dimension approximation that corresponds to the size of a circular region with the same area. It is computed as $\sqrt{\left(4*Area/\pi \right)}$.
- *Solidity*: this is a metric that quantifies the compactness of the trajectory region by considering the relationship between its area and the area of its convex hull. It provides insights into how tightly packed the trajectory is within its convex hull, and is computed as Area/ConvexArea.
- *Extent*: this quantifies the spatial occupancy of the trajectory region within its bounding box. It provides insights into how much space the trajectory occupies within its bounding box.
- *MaxIntensity*: the maximum intensity value of pixels within the trajectory region. It represents the highest brightness level present in the trajectory region.
- *MeanIntensity*: the average intensity value of all pixels within the trajectory region. It provides a measure of the overall brightness.
- *MinIntensity*: the minimum intensity value of pixels within the trajectory region. It represents the darkest region of the trajectory region.

SURF is a fast and robust feature detection algorithm in image processing and computer vision, designed for efficiently identifying key points in images [30]. It achieves speed by using box filters and integral images to approximate convolutions, making it efficient for real-time applications. It handles scale and rotation changes, extending the scale-invariant feature transform (SIFT) algorithm. Indeed, by using a scale-space representation and approximating the Hessian matrix determinant, SURF tries to identify points that are invariant to changes in scale and rotation [34].

SURF works by selecting interest points where significant changes occur in intensity or color. These interest points are locations where the algorithm focuses its attention for further analysis. Once interest points are identified, SURF describes the local neighborhoods around these points. It does this by considering image gradients in horizontal and vertical directions, capturing information about the local structure of the image. Then it creates unique descriptors for each interest point, based on the information in its local neighborhood. These descriptors are designed to be distinctive and invariant to various image transformations [30].

The robust feature detection capability of SURF seamlessly integrates into our trajectory image analysis through the use of the ‘bagOfFeatures‘ function in MATLAB R2023a, leveraging RGB trajectory images. By employing the ‘Detector’ option for ‘PointSelection’, the function meticulously selects point locations using a SURF ‘Detector’ with the ‘PointSelection’ property. This ensures thorough selection of feature points through an optimized SURF detector, enhancing precision and efficiency in the subsequent feature extraction process. The function’s outcome is a bag of visual words, encapsulating a variety of distinctive features in the trajectory image. These features may include descriptors resembling ‘straight’, ‘curve’, or ‘circular’, each contributing to the detailed and varied representation of the trajectory pattern. Subsequently, employing the ‘encode’ function with the bag of visual words as input yields a feature vector, which is essentially forming a histogram capturing the occurrences of visual words in the trajectory image (note: https://it.mathworks.com/help/vision/ref/bagoffeatures.html (accessed on 10 October 2023)).

The histogram for each image effectively captures the unique characteristics of the movement pattern within the smart-home environment.

#### 2.4.2. Synthetic Minority Oversampling (SMOTE)

In this study, we observe a significant imbalance in trajectory data, as delineated in the Smart-Home Infrastructure, i.e., the subset of the CASAS dataset, encompassing 80 CH older adults and a more constrained set of 19 PwDs. In this regard, the distribution of trajectories per person for CH and PwD is reported in Table 4. The standard deviation (STD) functions as an indicator of the variability in trajectory numbers among individuals. Given the uneven walking patterns of individuals within the smart-home, the number of trajectory images per person fluctuates. Notably, with an increase in the time interval of the segment (from 60 s to 480 s), there is a corresponding decrease in the number of trajectories for each individual.

The total number of samples for trajectories based on different time interval segmentations is as follows: 1629 in 60 s, 812 in 120 s, 381 in 240 s, and 208 in 480 s.

To address the imbalance problem effectively, we integrate SMOTE, a method categorized under data augmentation. SMOTE assumes a pivotal role in rectifying the dataset’s imbalance by generating synthetic feature vectors. The process involves selecting instances that closely align in the feature space, establishing connections between them, and subsequently generating new samples along these connections [31]. To clarify, an “instance” here represents a single trajectory data point, capturing the movement pattern of an individual. In this scenario, “samples” would be subsets of these instances, comprising trajectories from CH older adults or PwDs.

The SMOTE procedure begins by randomly selecting an instance, denoted as *a*, from the minority class. Subsequently, the *k* nearest minority neighbors of *a* are identified, where k=5 in our case. A synthetic instance is then created by randomly selecting one of these neighbors, referred to as *b*, and connecting *a* and *b* to form a line segment in the feature space. The resulting synthetic instances are effectively generated as convex combinations of the chosen instances *a* and *b* [35].

The strategic application of SMOTE proves notably impactful in the domain of cognitive assessment, especially when dealing with feature vectors extracted from trajectory images. As previously mentioned, augmenting instances enhances the representation of trajectories from the minority class, resulting in a more balanced and robust dataset for our cognitive assessment framework.

#### 2.4.3. Short-Term Cognitive Assessment

In this sub-module of our functional prototype of the system architecture, we evaluate different ML classifiers designed to categorize movement patterns as either walked by a CH subject or a PwD, based on the predicted category. These ML classifiers are:

- Naïve Bayes (NB);
- *k*-nearest neighbor (kNN), where k=5;
- Decision tree (DT);
- Support vector machine (SVM) with ‘rbf’ kernel and regularization parameter equal to 0.7;
- Neural network (NN), composed of 100 neurons in a hidden layer, ReLU activation function, Adam optimizer, and trained for 1000 epochs, with a batch size equal to 200.

The short-term cognitive assessment evaluates each path traversed by the resident as either “0—normal” or “1—abnormal”. In this context, normal trajectories mirror the walking patterns of CH individuals, while abnormal trajectories resemble those observed in PwDs. Our approach involves collaborative training, where the cloud-based model training receives training sets with tagged feature vectors for locomotion, and various instances of designated models locally assimilate and train on these sets. This sub-module further refines the short-term cognitive assessment based on these features. For privacy reasons, the feature vectors are processed solely by the trusted cloud residing in cloud-based model training, ensuring data security.

#### 2.4.4. Long-Term Cognitive Assessment

The Long-Term Cognitive Assessment sub-module employs ML algorithm predictions to generate a diagnostic hypothesis regarding the subject’s cognitive health. For robust results, the sub-module requires data spanning an extended period, such as the preceding 30 days. This time frame ensures a comprehensive examination of the smart-home resident’s movements. However, in our study, the dataset, comprising information from 99 individuals, was limited in its observational scope. Each person was monitored for only a few hours on a single day, presenting a challenge to achieving a more extended and in-depth analysis.

The long-term prediction, ltp, is determined by the most frequently predicted class using the formula:

$$ltp=\underset{c\in \{‘\mathrm{CH}’,‘\mathrm{PwD}’\}}{arg max}\;|\;\left\{c\in \left\{c_{1},\ldots ,c_{m}\right\}\right\}\;|,$$

where $c_{1},\ldots ,c_{m}$ represents the history of short-term cognitive assessments for the smart-home resident’s movement patterns.

## 3. Experimental Evaluation

In this section, we present the development and experimental evaluation of our proposed functional prototype of the system architecture.

### 3.1. Setup

Our functional prototype of the system architecture was implemented in Python, and the Image Processing Toolbox in MATLAB was employed for extracting CRF and SURF. The experiments were conducted on a MacBook M1 Pro. Our approach involves short-term and long-term cognitive assessment of movement patterns, employing a leave-one-person-out (LOPO) cross-validation strategy. In LOPO, one resident’s trajectory data are exclusively used for testing, ensuring that each resident’s data are never simultaneously used for both training and testing.

Performance assessment relies on weighted and macro-averaged precision, recall, and $F_{1}$-score. These scores are defined as follows:

(1) $$Precision=\frac{TP}{TP+FP},$$

(2) $$Recall=\frac{TP}{TP+FN},$$

(3) $$F_{1}\mathrm{-score}=\frac{2*Precision*Recall}{Precision+Recall},$$

where TP represents the true positive rate, FP denotes the false positive rate, and FN is equivalent to the false negative rate. Precision quantifies the count of items accurately labeled as part of the positive class, divided by the total items correctly or incorrectly labeled within the same class. Recall assesses the ratio of correctly identified positives (also known as TP). Lastly, the $F_{1}$-score conveys the balance between the precision and the recall.

Additionally, the macro-averaged scores are well-suited for handling imbalanced datasets, with the macro $F_{1}$-score offering robustness in cases of imbalanced data by treating all classes equally, regardless of their sizes. In this series of experiments, we assessed our visual encoding method presented in Section 2.3.2, along with the connectivity and descriptor-based features introduced in Section 2.4.1, using traditional ML classifiers, which reside in the short-term cognitive assessment sub-module and are presented in Section 2.4.3.

Building upon Zolfaghari et al.’s prior investigation [26], we found that the integration of heterogeneous features enhances recognition accuracy. Furthermore, our goal was to develop a thorough depiction of trajectory images through the integration of these characteristics. This depiction can be applied to detect atypical movement patterns within smart-home settings.

Therefore, we conducted the first set of analyses, relying solely on the combination of the two feature categories, CRF and SURF, extracted by the connectivity and descriptor-based feature extraction sub-module in the cognitive assessment module from encoded trajectory images. In the second set of evaluations, we applied SMOTE to address the imbalanced dataset, aiming to enhance the ML models’ performance and adaptability in the imbalance situation. This technique is explained in Section 2.4.2. The last set of experiments pertains to long-term cognitive assessment, as described in Section 2.4.4.

### 3.2. Experimental Results

In the following, we report the results of our experimental evaluation.

#### 3.2.1. Short-Term Cognitive Assessment by Integrating CRF and SURF

**Figure 4:**
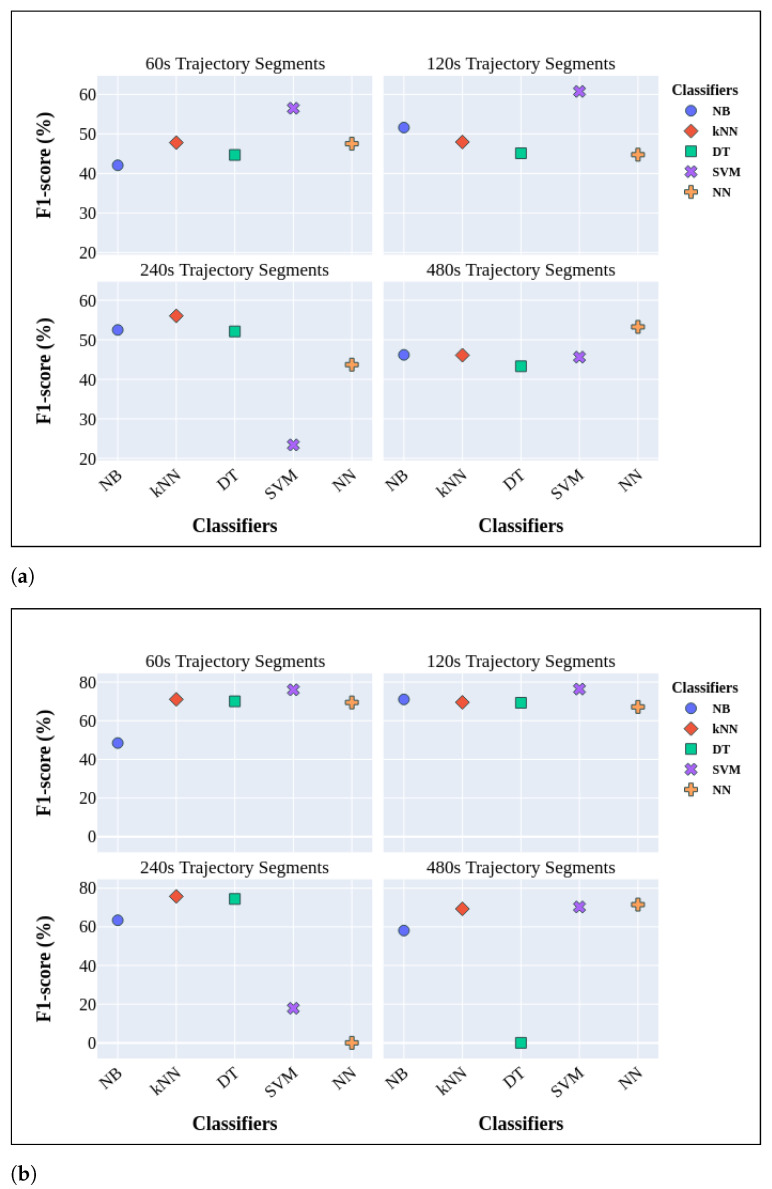
Macro-averaged $F_{1}$-scores (**a**) and weighted-averaged $F_{1}$-scores (**b**) for cognitive assessment by employing traditional models and LOPO cross-validation, taking into account both CRF and SURF.

The results based on utilization of CRF and SURF is reported in Figure 4. Among various ML models, SVM demonstrated superior performance, with macro-averaged $F_{1}$-scores of 60.75% and weighted-averaged $F_{1}$-scores of 76.39% for the trajectory segments of $T_{s}=120$ s. However, the SVM model’s performance declined noticeably with an increased time interval, reaching a weighted-averaged score of 17.86% and a macro-averaged score of 23.39% for the $T_{s}=240$ s trajectory segments. Furthermore, within distinct time intervals, it was noted that kNN consistently yielded robust results in both macro-averaged and weighted-averaged scores. Notably, there were no significant fluctuations in the results across different time intervals. NB achieved its lowest macro-averaged $F_{1}$-score in $T_{s}=60$ s segments, while DT and NN exhibited their respective lowest scores in the $T_{s}=120$ s segments. Similarly, SVM’s lowest score was noted in the $T_{s}=240$ s segments, and DT performed least effectively in the $T_{s}=480$ s segments. These outcomes indicate the limitations of these models in successfully identifying a noteworthy portion of minority class trajectories, particularly those linked to PwDs, especially as the time interval increased.

Based on these findings, it becomes apparent that incorporating additional visual cues in encoded trajectory images derived from sensor activations can lead to performance levels that are nearly satisfactory in the context of cognitive assessment applications.

Furthermore, Figure 4 highlights that, as the time interval between consecutive sensor activations indicating non-locomotion phases increases, there is a noticeable decline in the models’ performance, particularly in relation to the macro-averaged $F_{1}$-score. This decline can be attributed to the increased complexity of trajectories over longer time intervals, posing challenges in extracting meaningful features for accurate classification and cognitive assessment. Additionally, the observed results in the weighted-averaged $F_{1}$-scores are notably superior to those in the macro-averaged $F_{1}$-scores. This discrepancy is a consequence of class imbalance, wherein the classifiers exhibit a bias toward predicting the more prevalent class, CH.

#### 3.2.2. Short-Term Cognitive Assessment by Applying SMOTE

In order to handle the imbalance problem we mentioned, in this series of experiments we employed the Synthetic Minority Oversampling (SMOTE) technique. Following feature extraction, additional feature vectors were generated by synthesizing new instances from existing ones. The results of these experiments are presented in Table 5.

Notably, there is an improvement in the performance of almost all models in terms of both macro-averaged and weighted-averaged results in relation to the analysis presented in Section 3.2.1.

Among the evaluated models, SVM demonstrated the best results in the $T_{s}=60$ s trajectory segments, achieving 70.20% and 82.88% $F_{1}$-scores in macro-averaged and weighted-averaged, respectively. Conversely, NB yielded the lowest result in the $T_{s}=60$ s trajectory segments, obtaining 43.85% in the macro-averaged $F_{1}$-score and 51.53% in the weighted-averaged $F_{1}$-score, respectively. However, as the time interval between consecutive sensor activations increased, SVM shows the lowest performance in the $T_{s}=480$ s trajectory segments, with a 13.78% weighted-averaged $F_{1}$-score and a 13.03% macro-averaged $F_{1}$-score. When examining kNN and NN, the outcomes exhibited minimal variation with increasing time intervals. In contrast, DT consistently demonstrated improved results, particularly in macro-averaged and weighted-averaged metrics, as the time intervals increased.

A noteworthy observation emerges when comparing kNN and NN results with and without employing SMOTE. Notably, without SMOTE, kNN achieved a 56.04% and 75.74% $F_{1}$-score in macro-averaged and weighted-averaged categories, respectively. NN gained a 53.25% and 71.50% $F_{1}$-score in macro-averaged and weighted-averaged categories, respectively, without SMOTE. This comparison underscores the impact of employing SMOTE in enhancing the performance metrics of kNN and NN. This behavior could be attributed to the complexity introduced in decision boundaries by generating synthetic samples. Additionally, the sensitivity of kNN to its parameters, including the selection of a distance metric and the number of neighbors, might play a role. The incorporation of synthetic samples through SMOTE could potentially impact the optimal parameter settings for kNN, contributing to the observed behavior.

#### 3.2.3. Comparative Analysis of the Model vs. State-of-the-Art

We performed a comprehensive comparative analysis that included our proposed approach, referred to as CRF + SURF + SMOTE. This approach integrates CRF and SURF, enhancing the representation of the minority class, which in this case pertains to PwDs, through the application of SMOTE. We benchmarked our approach against established models found in the existing literature. Given that the most favorable outcomes were obtained in segments with $T_{s}=60$ s time intervals, these results were utilized for comparison against the achievements of state-of-the-art approaches. The results are presented in Table 6, and the evaluation is conducted using LOPO cross-validation.

To facilitate this comparison, we introduced a baseline feature extraction method, labeled as *Numeric Trajectory Features* (NTF). This method involves the extraction of features aligned with clinical indicators of cognitive decline found in the literature. The computation of spatio-temporal features, such as trajectory length, duration, centroids, and the number of activations for each position sensor along the trajectory, as well as Martino-Saltzman indicators [17] and low-level motion indicators (overall jerk, sharp angles, and overall straightness), relies on algorithms presented in [9] for the recognition of cognitive issues.

Furthermore, we compare the technique proposed in this work with *Randomly Rotated Spatio-Temporal Features* (RR-STF) in [26]. This method adopted an approach that combined spatio-temporal features with features extracted from images depicting trajectories within a smart-home. The authors introduced a data augmentation technique based on random image rotation to enhance the generalization of the trained model.

It is worth noting that we implemented these methods by referencing the information presented in their respective papers, which detailed the system architecture. By reviewing the results, it is evident that our CRF + SURF + SMOTE approach gained better results compared with NTF and RR-STF [26]. The classifier achieving the best performance in this pool of experiments is SVM, with a $F_{1}$-score of 70.20%.

#### 3.2.4. Long-Term Cognitive Assessment Results

**Figure 5:**
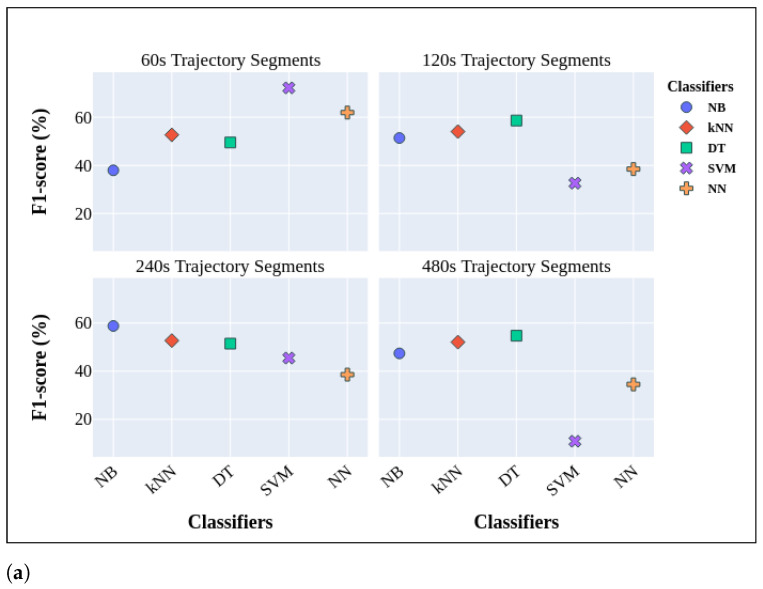

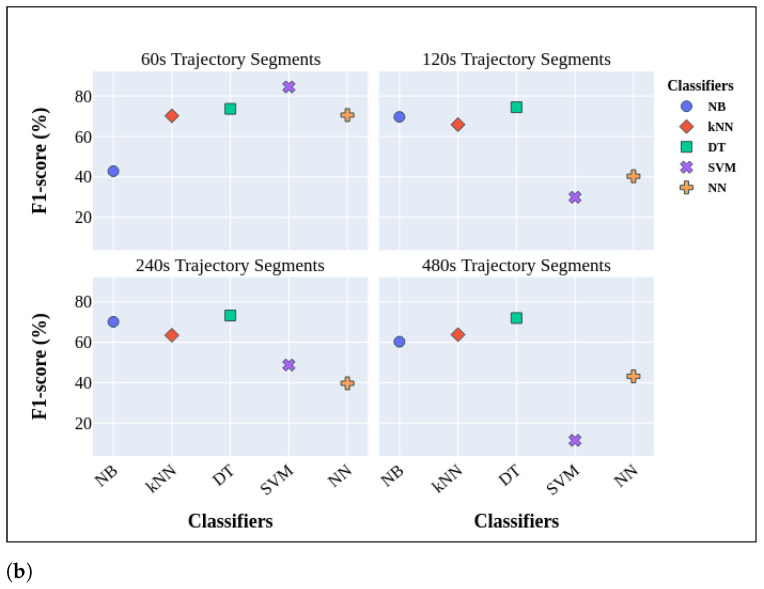
Macro-averaged (**a**) and weighted-averaged measures (**b**) for long-term cognitive assessment.

In this evaluation, we apply the algorithm for long-term cognitive assessment described in Section 2.4.4 on the proposed functional prototype of the system architecture utilizing CRF, SURF, and SMOTE. The results are summarized in Figure 5. Remarkably, as anticipated, SVM stands out among various machine learning algorithms, delivering highly promising outcomes with $T_{s}=60$ s. It achieves a macro-averaged $F_{1}$-score of 72.22% and a weighted-averaged $F_{1}$-score of 84.51%. This performance is particularly noteworthy for the short-term cognitive assessment, as SVM consistently produces the best results in this context.

Additionally, this performance signifies a substantial improvement, showing a 10% increase in the macro-averaged $F_{1}$-score and a 5% increase in the weighted-averaged $F_{1}$-score compared with the previous work presented in [26]. In that work, the achieved scores were 62.56% for the macro-averaged $F_{1}$-score and 79.1% for the weighted-averaged $F_{1}$-score. However, upon evaluating the NN performance in long-term cognitive assessment, it is evident that its performance is comparable to the one of other algorithms with $T_{s}=60$ s, and it could gain 61.94% for the macro-averaged $F_{1}$-score and 70.59% for the weighted-averaged $F_{1}$-score. In this analysis, depicted in Figure 5, the stability in the performance of DT and kNN stands out, as opposed to NB or SVM. However, it is crucial to highlight that both DT and kNN exhibited poor performance compared with SVM, particularly concerning $T_{s}=60$ s. This observation implies a reduced level of effectiveness for DT and kNN in this particular context. However, it is worth noting that DT, in particular, performed relatively well compared with NB and kNN.

## 4. Discussion and Conclusions

This study tackles the challenge of cognitive assessment through the analysis of indoor movements. Our methodology entails the extraction of visual cues from trajectories, taking into account sensor activations, their position and direction, object interactions, CRF and SURF, which are well-known features in computer vision tasks, the application of SMOTE, and the incorporation of classical ML algorithms. The results, based on a real-world dataset of CH older adults and PwDs, show promising short-term and long-term cognitive assessment accuracy. Our experimental evaluation of the proposed functional prototype of the system architecture highlights the superior performance achieved through the integration of our visual cues, coupled with the utilization of features extracted using CRF and SURF and SMOTE. The efficacy of our approach is attributed to the inclusion of supplementary features, such as speed, position, direction, object interactions, and low-level movement indicators. These elements, not captured by existing solutions, contribute to the strength and comprehensiveness of our approach.

Additionally, the experiments demonstrate a substantial enhancement in the assessment performance of the proposed functional prototype of the system architecture when considering the entire trajectory history. It is crucial to note that, despite the dataset including over 99 individuals, each person was observed for only a few hours on a single day. This limited observation period may not provide sufficient data to predict the cognitive status of all individuals reliably. Consequently, we anticipate achieving more accurate predictions by incorporating a longer history of observations. However, this intuitive assumption requires validation through additional experiments conducted in a larger trial. Indeed, the obtained macro-averaged $F_{1}$-score indicates that the proposed functional prototype of the system architecture could potentially serve as valuable assistance for clinicians in conducting a clinical evaluation of the cognitive health status of elderly individuals. Nevertheless, to substantiate this hypothesis, a comprehensive trial involving the collaboration of clinicians and the deployment of our system in real-world conditions is imperative. Moreover, considering the challenge of limited large-scale datasets in sensor-based trajectory data mining, our results highlight the efficacy of incorporating additional visual cues via an encoded trajectory image method, extracting features by CRF and SURF, and enhancing them with SMOTE. This not only encodes rich information from sensor events but also improves model generalization, leading to enhanced performance. This approach proves well-suited for applications in healthcare and sensor-rich smart environments.

Nevertheless, several challenges persist. While SMOTE effectively generates synthetic minority class instances, it has limitations and potential biases. These include overfitting to the minority class, inaccurate representation of the true distribution, and sensitivity to noisy instances. To address these concerns, researchers are exploring alternatives like cost-sensitive learning, ensemble methods, and various resampling techniques. This flexibility allows tailoring the approach to specific dataset challenges, mitigating potential biases introduced by a singular technique like SMOTE. Moreover, the results presented in Figure 4 and Figure 5 and Table 5 consistently show that weighted-averaged values outperform macro-averaged ones in ML algorithms. This bias is attributed to the model’s inclination to predict the most frequent class, which, in our context, is CH.

As previously stated, our study assumed a single resident in the smart-home, providing a focused analysis but introducing a limitation. Addressing this limitation could be a focus of future research by exploring the integration of an identity-aware indoor localization system. Such a system could enable the distinction of movement traces and interactions among multiple smart-home residents. Additionally, the implementation of an algorithm for multi-resident data association, as proposed by Riboni et al. [32], could further enhance the overall approach. Moreover, deploying a smart-home system with PIR motion sensors, door sensors, and RFID-embedded sensors is indeed feasible, emphasizing compatibility, ease of integration, cost-effectiveness, user acceptance, scalability, and interoperability with existing devices as pivotal considerations. Additionally, factors such as power consumption, customization flexibility, adherence to regulations, availability of technical support, and the implementation of robust security measures are essential for a successful deployment.

The implementation of sensor-based AI systems determines different concerns in terms of privacy and ethics considerations, especially when those systems are deployed at patients’ homes. Indeed, smart-home systems acquire and process very sensitive data such as locomotion, sleep patterns, presence/absence of people in the home, activities of daily living, and personal routines. Hence, a privacy-by-design methodology should be employed to implement these systems, possibly anonymizing the data and executing reasoning at the edge in order to limit the release of data to third parties. Strong security measures are also necessary to prevent unauthorized access to sensitive information. When those systems are addressed to people with cognitive decline, a critical issue is acquiring informed consent from inhabitants. Indeed, some people with cognitive impairment cannot fully understand the research objectives or the privacy implications regarding personal data acquisition. Hence, it is necessary to rely on statutory frameworks for assessing the decision-making capacity of inhabitants and to ensure the ethical deployment of the pervasive healthcare platform [36].

Furthermore, there are significant issues regard the acceptance of AI diagnostic systems in clinical settings [37]. In order to mitigate these issues, it is important to involve the stakeholders, including caregivers and healthcare professionals, in every step of the pervasive healthcare system design and implementation. The significance of conducting pilot tests in diverse smart-home environments cannot be overstated; this approach helps in identifying and addressing potential challenges, ensuring the system’s feasibility and optimizing the user experience before embarking on widespread deployment. Also, we believe that leveraging advanced transfer learning methods tailored for image classification could potentially facilitate the portability of training data [38]. However, confirmation of this aspect awaits additional experiments with diverse datasets.

Moreover, our experimental evaluation involved employing a dataset containing a diverse group of subjects, with each individual’s behavior monitored in a controlled environment. While this approach is valuable for controlled experiments, it has inherent limitations in capturing the full spectrum of naturalistic behaviors [39]. Real-world indoor movements encompass various variables, such as activity variations, ambient conditions, and obstacles, which can significantly impact the system’s classification performance. Additionally, a noteworthy challenge in our study was the presence of an unusually high number of sensors within the smart-home setting. It is important to acknowledge that such sensor-rich environments may not be representative of the typical smart-homes found in the real world. This unique characteristic poses an additional challenge that needs to be considered in the interpretation and generalization of our findings.

Furthermore, detecting abnormal movements in PwDs proved challenging due to limited data, highlighting the potential benefits of incorporating additional sensors, such as wearable sensors. Furthermore, our study assumed that the training data matched the older adult’s home environment. To overcome concerns related to data scarcity, we plan to implement advanced transfer learning methods and evaluate various data augmentation strategies. This approach will allow us to leverage training data from diverse environments, thereby enhancing the system’s adaptability and overall performance. In conclusion, our future endeavors focus on improving the system’s adaptability to multi-resident environments and conducting experiments in fully naturalistic settings for extended periods, all while carefully considering ethical, privacy, and security aspects. 

## Figures and Tables

**Table 1:** Raw data from discrete sensors.

Timestamp (*t*)	Sensor ID (s_id)	Value (*v*)
09:10:00.094833	M001	ON
09:10:01.014748	M023	ON
09:10:01.045917	M021	OFF
09:10:01.093183	M022	OFF
09:10:02.087933	M023	OFF
09:10:03.072194	M023	ON
09:10:05.014012	D012	OPEN
09:10:05.043057	M001	OFF
09:10:06.038858	M023	OFF
09:10:19.094168	D012	CLOSE

**Table 2:** Sensor position table.

Sensor ID (s_id)	*x*	*y*
M025	0.95	1.26
I012	1.07	8.78
I011	0.76	9.10
M024	2.99	1.21
D001	2.17	0.23
T003	3.02	4.67
M036	5.26	4.97
M030	5.81	6.06
D003	5.56	6.40
M029	5.84	7.16

**Table 3:** Considered colors and their corresponding category in the current article.

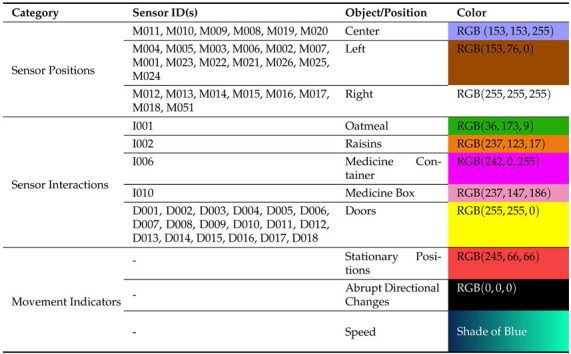

**Table 4:** Average number of trajectories per person, with their respective STDs.

Trajectory Segments	CH	PwD
60 s	12.87 ± 7.54	3.26 ± 7.08
120 s	6.34 ± 4.02	1.70 ± 3.82
240 s	3.04 ± 1.93	0.73 ± 1.68
480 s	1.64 ± 1.13	0.42 ± 0.99

**Table 5:** Movement images classification results using CRF and SURF extracted from encoded images and enriched by SMOTE. Bold numbers represent the best results for each experiment.

Segments	Measures (%)		NB	KNN	DT	SVM	NN
60 s	Weighted	Precision	68.71	68.10	68.96	**85.11**	64.63
		Recall	46.81	58.11	76.17	**85.39**	50.37
		$F_{1}$-score	51.53	61.80	71.41	**82.88**	55.21
	Macro	Precision	50.89	50.42	52.47	**84.40**	46.63
		Recall	51.35	50.60	51.03	**66.44**	44.85
		$F_{1}$-score	43.85	48.74	49.60	**70.20**	42.99
120 s	Weighted	Precision	**67.89**	67.54	66.92	65.35	65.99
		Recall	59.07	54.75	**59.93**	40.70	50.93
		$F_{1}$-score	62.32	58.82	**62.73**	44.44	55.41
	Macro	Precision	**51.59**	51.04	50.43	48.95	49.40
		Recall	**52.14**	51.53	50.57	48.56	49.10
		$F_{1}$-score	**50.28**	48.33	49.54	39.46	45.55
240 s	Weighted	Precision	73.34	71.41	71.66	**73.61**	71.57
		Recall	61.58	53.95	**74.74**	46.32	60.79
		$F_{1}$-score	65.38	58.73	**72.97**	50.60	64.54
	Macro	Precision	55.40	52.83	**55.43**	54.31	53.45
		Recall	**58.22**	54.51	54.09	56.43	55.17
		$F_{1}$-score	53.80	48.56	**54.34**	44.54	51.95
480 s	Weighted	Precision	68.16	69.97	**71.20**	28.08	66.98
		Recall	57.01	56.04	**73.43**	13.05	63.77
		$F_{1}$-score	60.95	60.31	**72.19**	13.78	65.25
	Macro	Precision	50.58	52.49	**55.96**	20.38	49.05
		Recall	50.85	53.79	**54.94**	18.84	48.88
		$F_{1}$-score	48.53	49.60	**55.23**	13.03	48.78

**Table 6:** Comparative analysis of the best result of the proposed technique against existing best results gained by literature approaches in terms of macro-averaged $F_{1}$-score. The bold approach highlight our recommended functional prototype for the system architecture, while bold numbers signify the optimal results attained.

Approaches	Measures (%)	NB	KNN	DT	SVM	NN
RR-STF	Precision	49.58	50.15	50.23	60.26	60.27
	Recall	49.04	50.17	50.4	60.26	62.85
	$F_{1}$-score	47.87	50.11	49.43	60.26	61.17
NTF	Precision	57.60	53.70	58.2	58.50	61.40
	Recall	55.60	56.80	63.60	58.80	73.40
	$F_{1}$-score	56.2	52.10	58.7	58.7	59.3
**CRF + SURF + SMOTE**	Precision	50.89	50.42	52.47	**84.40**	46.63
	Recall	51.35	50.6	51.10	**66.44**	44.85
	$F_{1}$-score	43.85	48.74	49.60	**70.20**	42.99

## Data Availability

The dataset used in this article can be downloaded at: http://casas.wsu.edu/datasets/assessmentdata.zip (accessed on 1 January 2020).

## References

[1] Gerland P., Hertog S., Wheldon M., Kantorova V., Gu D., Gonnella G., Williams I., Zeifman L., Bay G., Castanheira H. (2022). World Population Prospects 2022: Summary of Results.

[2] Rechel B., Grundy E., Robine J.M., Cylus J., Mackenbach J.P., Knai C., McKee M. (2013). Ageing in the European union. Lancet.

[3] Prince M., Prina M., Guerchet M. (2013). World Alzheimer Report 2013: Journey of Caring.

[4] Rashidi P., Mihailidis A. (2012). A survey on ambient-assisted living tools for older adults. IEEE J. Biomed. Health Inform..

[5] Zolfaghari S., Suravee S., Riboni D., Yordanova K. (2023). Sensor-Based Locomotion Data Mining for Supporting the Diagnosis of Neurodegenerative Disorders: A Survey. ACM Comput. Surv..

[6] Gochoo M., Tan T.H., Velusamy V., Liu S.H., Bayanduuren D., Huang S.C. (2017). Device-free non-privacy invasive classification of elderly travel patterns in a smart house using PIR sensors and DCNN. IEEE Sensors J..

[7] Amiribesheli M., Benmansour A., Bouchachia A. (2015). A review of smart homes in healthcare. J. Ambient Intell. Humaniz. Comput..

[8] Riboni D., Bettini C., Civitarese G., Janjua Z.H., Helaoui R. (2016). SmartFABER: Recognizing fine-grained abnormal behaviors for early detection of mild cognitive impairment. Artif. Intell. Med..

[9] Khodabandehloo E., Riboni D., Alimohammadi A. (2021). HealthXAI: Collaborative and explainable AI for supporting early diagnosis of cognitive decline. Future Gener. Comput. Syst..

[10] Sprint G., Cook D.J., Fritz R. (2020). Behavioral differences between subject groups identified using smart homes and change point detection. IEEE J. Biomed. Health Inform..

[11] Riboni D., Bettini C., Civitarese G., Janjua Z., Helaoui R. Fine-grained Recognition of Abnormal Behaviors for Early Detection of Mild Cognitive Impairment. Proceedings of the 2015 IEEE International Conference on Pervasive Computing and Communications (PerCom).

[12] Zolfaghari S., Khodabandehloo E., Riboni D. (2022). TraMiner: Vision-based analysis of locomotion traces for cognitive assessment in smart-homes. Cogn. Comput..

[13] Kumar A., Lau C.T., Ma M., Chan S., Kearns W. Trend Analysis in the Trajectory of the Dementia Patients. Proceedings of the ICSEC.

[14] Dodge H., Mattek N., Austin D., Hayes T., Kaye J. (2012). In-home walking speeds and variability trajectories associated with mild cognitive impairment. Neurology.

[15] Kearns W.D., Fozard J.L., Nams V.O., Craighead J.D. (2011). Wireless telesurveillance system for detecting dementia. Gerontechnology.

[16] Algase D.L., Moore D.H., Vandeweerd C., Gavin-Dreschnack D. (2007). Mapping the maze of terms and definitions in dementia-related wandering. Aging Ment. Health.

[17] Martino-Saltzman D., Blasch B.B., Morris R.D., McNeal L.W. (1991). Travel behavior of nursing home residents perceived as wanderers and nonwanderers. Gerontol..

[18] Khodabandehloo E., Alimohammadi A., Riboni D. (2022). FreeSia: A Cyber-physical System for Cognitive Assessment through Frequency-domain Indoor Locomotion Analysis. ACM Trans. Cyber-Phys. Syst. (TCPS).

[19] Lin Q., Zhang D., Huang X., Ni H., Zhou X. Detecting wandering behavior based on GPS traces for elders with dementia. Proceedings of the 12th International Conference on Control Automation Robotics & Vision.

[20] Ng J., Kong H. Not All Who Wander Are Lost: Smart Tracker for People with Dementia. Proceedings of the 2016 CHI Conference Extended Abstracts on Human Factors in Computing Systems.

[21] Vuong N., Chan S., Lau C.T., Lau K. Feasibility study of a real-time wandering detection algorithm for dementia patients. Proceedings of the First ACM MobiHoc Workshop on Pervasive Wireless Healthcare.

[22] Lin Q., Zhao W., Wang W. (2018). Detecting Dementia-Related Wandering Locomotion of Elders by Leveraging Active Infrared Sensors. J. Comput. Commun..

[23] Khodabandehloo E., Riboni D. (2020). Collaborative trajectory mining in smart-homes to support early diagnosis of cognitive decline. IEEE Trans. Emerg. Top. Comput..

[24] Faruk T., Shum L.C., Iaboni A., Khan S.S. (2023). Walking path images from real-time location data predict degree of cognitive impairment. Artif. Intell. Med..

[25] Zolfaghari S., Khodabandehloo E., Riboni D. Towards vision-based analysis of indoor trajectories for cognitive assessment. Proceedings of the 2020 IEEE International Conference on Smart Computing (SMARTCOMP).

[26] Zolfaghari S., Loddo A., Pes B., Riboni D. A combination of visual and temporal trajectory features for cognitive assessment in smart home. Proceedings of the 2022 23rd IEEE International Conference on Mobile Data Management (MDM).

[27] Zolfaghari S., Massa S.M., Riboni D. (2023). Activity Recognition in Smart Homes via Feature-Rich Visual Extraction of Locomotion Traces. Electronics.

[28] Vuong N.K., Chan S., Lau C.T. (2014). Automated detection of wandering patterns in people with dementia. Gerontechnology.

[29] Kong C., Gao J., Zhu J., Ehn A., Aldén M., Li Z. (2017). Characterization of an AC glow-type gliding arc discharge in atmospheric air with a current-voltage lumped model. Phys. Plasmas.

[30] Bay H., Tuytelaars T., Van Gool L. Surf: Speeded up robust features. Proceedings of the Computer Vision–ECCV 2006: 9th European Conference on Computer Vision.

[31] Chawla N.V., Bowyer K.W., Hall L.O., Kegelmeyer W.P. (2002). SMOTE: Synthetic minority over-sampling technique. J. Artif. Intell. Res..

[32] Riboni D., Murru F. (2020). Unsupervised recognition of multi-resident activities in smart-homes. IEEE Access.

[33] Cook D.J., Schmitter-Edgecombe M., Crandall A., Sanders C., Thomas B. Collecting and disseminating smart home sensor data in the CASAS project. Proceedings of the CHI Workshop on Developing Shared Home Behavior Datasets to Advance HCI and Ubiquitous Computing Research.

[34] Lowe D.G. (2004). Distinctive image features from scale-invariant keypoints. Int. J. Comput. Vis..

[35] He H., Ma Y. (2013). Imbalanced Learning: Foundations, Algorithms, and Applications.

[36] Alzheimer Europe (2023). Dementia in Europe Yearbook 2023: Legal Capacity and Supported Decision-Making Related to Dementia.

[37] Han C., Rundo L., Murao K., Nemoto T., Nakayama H. Bridging the gap between AI and healthcare sides: Towards developing clinically relevant AI-powered diagnosis systems. Proceedings of the IFIP International Conference on Artificial Intelligence Applications and Innovations.

[38] Deng C., Xue Y., Liu X., Li C., Tao D. (2018). Active transfer learning network: A unified deep joint spectral–spatial feature learning model for hyperspectral image classification. IEEE Trans. Geosci. Remote Sens..

[39] De-La-Hoz-Franco E., Ariza-Colpas P., Quero J.M., Espinilla M. (2018). Sensor-based datasets for human activity recognition—A systematic review of literature. IEEE Access.

